# ABO blood types may affect transient neurological events after surgical revascularization in patients with moyamoya disease: a retrospective single center study

**DOI:** 10.1186/s12871-023-02385-6

**Published:** 2023-12-19

**Authors:** Mei-Ping Qian, Mei-Rong Dong, Ming-Ming Han, Juan Li, Fang Kang

**Affiliations:** Department of Anesthesiology, Division of Life Sciences and Medicine, The First Affiliated Hospital of USTC, University of Science and Technology of China, Hefei, 230036 Anhui China

**Keywords:** Moyamoya Disease, ABO blood types, Postoperative transient neurological events

## Abstract

**Background:**

Moyamoya disease (MMD) is a cerebrovascular disease with unknown cause. Patients with MMD disease usually experience transient neurological events (TNEs) after revascularization surgery. This retrospective single-center study was aimed to explore the risk factors of postoperative TNEs after surgical revascularization in patients with MMD.

**Methods:**

We selected 324 patients who underwent surgical revascularization between January 2017 and September 2022 in our center. The perioperative characteristics of the patients were recorded and the outcome was TNEs after surgery. An analysis of risk factors contributing to postoperative TNEs by using logistic regression model.

**Results:**

Three hundred twelve patients were enrolled, and the incidence of postoperative TNEs was 34% in our study. Males were more likely to suffer from postoperative TNEs (OR = 2.344, *p* = 0.002). Preoperative ischemic presentation (OR = 1.849, *p* = 0.048) and intraoperative hypotension (OR = 2.332, *p* = 0.002) were associated with postoperative TNEs. Compared to patients with blood type O, patients with blood type A (OR = 2.325, *p* = 0.028), B (OR = 2.239, *p* = 0.027) and AB (OR = 2.938, *p* = 0.019) had a significantly higher incidence of postoperative TNEs. A risk prediction model for postoperative TNEs was established, and the established risk prediction area under the receiver operating characteristic curve (ROC) of the model was 0.741.

**Conclusions:**

Males, preoperative ischemic presentation and intraoperative hypotension were associated with postoperative TNEs. We also found a possible link between postoperative TNEs and ABO blood types after surgical revascularization for moyamoya patients.

## Background

Moyamoya disease (MMD) is a cerebrovascular disease with unknown cause. It is characterized by occlusion of the end and main branches of the internal carotid artery and often presents as intracranial hemorrhage, transient ischemic attacks or seizures [1, 2]. Compared with other treatments, surgical revascularization is the most effective treatment for MMD [3]. However, postoperative transient neurological events (TNEs) are usually occurred after surgical revascularization for MMD. Previous studies have indicated that the incidence of postoperative TNEs ranges from 15–67.1% [4, 5].

Postoperative complications are closely related to operation and anesthesia. Perioperative factors can affect intracranial hemodynamics in MMD patients, leading to new neurological complications in some patients after surgery [6]. The ABO blood group system is one of the most important systems known in humans that is used in clinical transfusion [7]. Previous research has shown that ABO blood types are correlated with many clinical conditions, including pain perception, diverse cancers, and cardiovascular diseases [8–10]. In a study [11] on the relationship between ABO blood type and delayed cerebral ischemia onset after aneurysmal subarachnoid hemorrhage, Researchers have found that blood type may be associated with cerebral vasospasm. Bypass vessels vasospasm can affect the perfusion of brain tissue and may lead to the occurrence of postoperative TNEs in patients with moyamoya disease. However, the relationship between blood type and TNEs after revascularization surgery for patients with MMD has not been reported. The aim of this retrospective single-center study was to identify the risk factors of postoperative TNEs after surgical revascularization in patients with MMD.

## Materials and methods

### Ethics and patients

This study was permitted by the Clinical Research Ethics Committee of The First Affiliated Hospital of University of Science and Technology of China, Anhui, China (2022-RE-352, Chairperson Prof. Zuojun Shen). Due to the non-interventional study design, the Institutional Review Board of The First Affiliated Hospital of University of Science and Technology of China waived the need for written informed consent from participants. This study was performed in accordance with the Declaration of Helsinki and with the STROBE Statement. The revascularization surgery was performed by the same neurosurgeon in our center between January 2017 and September 2022. Only patients with a confirmed diagnosis of MMD by preoperative diagnostic cerebral angiography were included in this study. We retrospectively reviewed the medical records of patients with Moyamoya disease older than 18 years who underwent a combined bypass of superficial temporal artery-middle cerebral artery (STA-MCA) anastomosis and encephalo-duro-arterio-synangiosis. Patients with missing data were excluded from the study.

### Methods and data acquisition

After entering the room, the patient underwent invasive arterial pressure measurement. All patients underwent general anesthesia with midazolam (0.02 mg/kg), etomidate (0.2 mg/kg), sufentanil (0.5 µg/kg), and cisatracurium (0.2 mg/kg) for induction and propofol (6 to 8 mg/kg/h), remifentanil (0.1–0.3 µg/kg/min) and sevoflurane (up to 0.5MAC) for maintenance. During the operation, the blood pressure was maintained above the preoperatively measured blood pressure (vasoactive drugs were used when necessary). A normal CO2 level (35–45 mm Hg) was maintained throughout surgery. Data were obtained by retrospectively reviewing records maintained in our institution. Patient demographic information, preoperative comorbidities, clinical features, preoperative imaging, preoperative laboratory data, intraoperative factors, postoperative results and laboratory data were recorded. The outcome was the occurrence of postoperative TNEs. The evaluation period for TNEs was the first 24 h postoperatively. Postoperative TNEs were defined as the appearance of transient neurological symptoms (lasting < 24 h) as previous literature [12, 13]. The episodes of neurological dysfunction as follows: (1) no sign of cerebral infarction or acute cerebral hemorrage in radiological images; (2) any reversible neurological defects (e.g., number) recognized subjectively by the patients; (3) any reversible neurological defects (e.g., hemiparesis, dysarthria) observed objectively by the doctors.

### Postoperative management and clinical follow-up

After extubation, patients were transferred to the neurosurgery intensive care unit (NICU). After the operation, blood pressure was maintained at the baseline level. Postoperative CT scan was routinely performed to identify hemorrhagic or infarction on the first postoperative day. Perform head CT-CTA assessment on patients with postoperative TNEs to rule out hemorrhagic events and confirm bypass patency. All patients should avoid the occurrence of hypovolemia and hypotension, and aspirin was administered for patients with ischemic-type MMD at the first day after surgery. Patients were given improve circulation drugs and resisting vasospasm drugs to relieve the symptoms of postoperative TNEs. Patients were clinically evaluated at discharge and after three months follow-up. Clinical follow up angiography was conducted 3 months postoperatively to evaluate the patency of the bypass vessel.

### Statistical analysis

The normal distribution test was checked in the continuous variables using Shapiro test. Continuous variables are expressed as the mean ± SD, and categorical variables are reported as counts with percentages. T-test or Mann‒Whitney test was used to test for differences between continuous variables. Chi-square test or Fisher’s exact test was used to compare the proportions of categorical variables between groups. Based on various predictive variables, univariate and multivariable logistic regressions were performed with postoperative TNEs as the outcome variable. A predictor variable was included in the multivariable model if *p* < 0.15. Statistically significant differences between groups were indicated by a *P* value < 0.05. The receiver operating characteristic (ROC) curve and the area under the curve (AUC) were calculated.

All significance tests were two-sided and *p* value less than 0.05 was considered as statistically significant. Tukey method was used for post-hoc multiple comparisons testing when needed. SAS version 9.4 (SAS Institute Inc.) was used for all analyses.

## Results

**Fig. 1:**
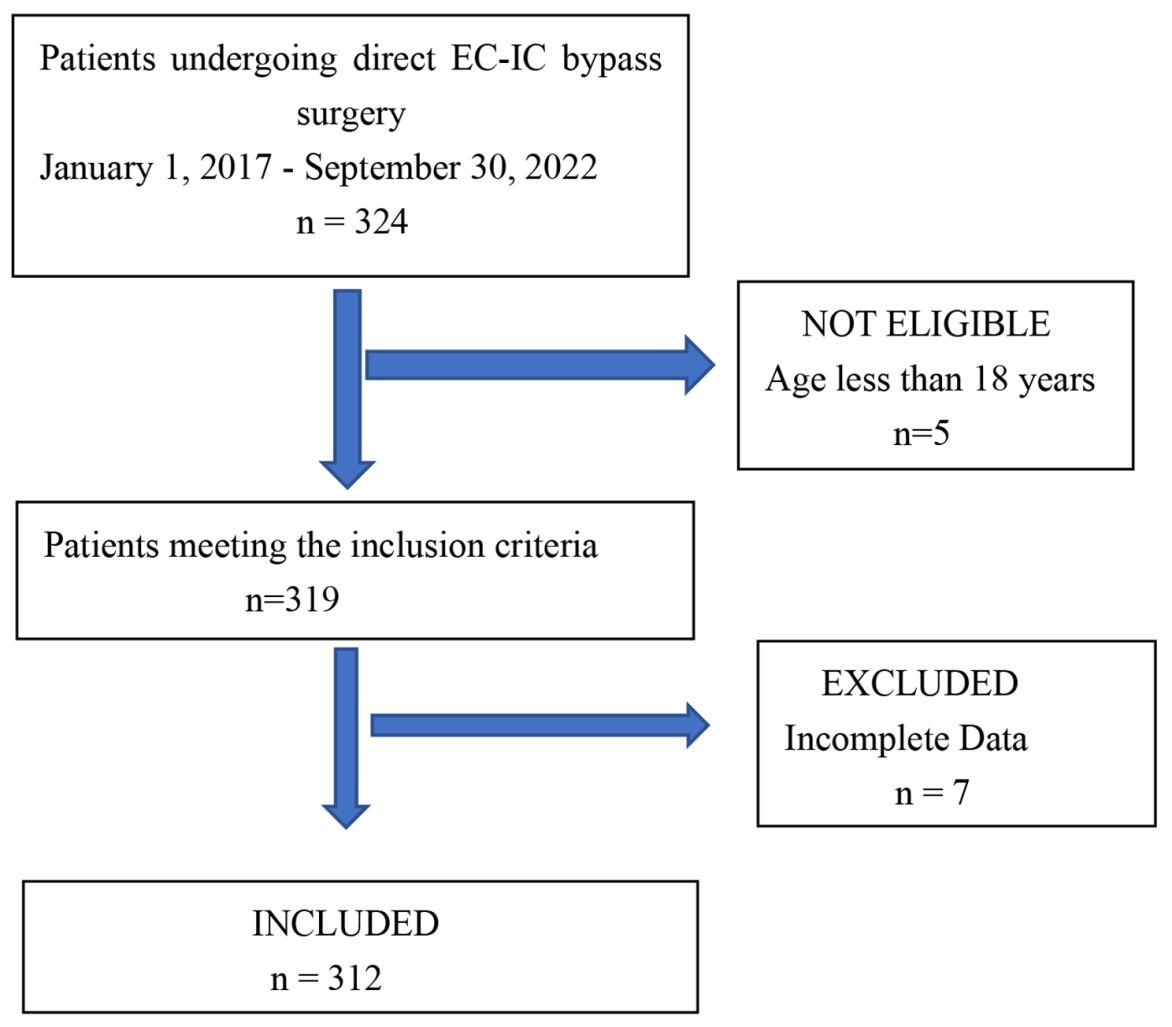
Study flow chart

Three hundred twenty-four patients who underwent unilateral surgical revascularization were included in this study, and 12 patients were excluded (Fig. 1). The incidence of postoperative TNEs was 34% (n = 106). Table 1 shows the perioperative characteristics of the patients in each group. There was significantly statistical difference between the two groups in the age, proportion of male patients, preoperative hemoglobin, fasting blood glucose, preoperative stroke, onset symptoms, intraoperative hypotension (relative decrease in systolic blood pressure more than 20% from baseline) and postoperative hemoglobin (*p* < 0.05).

**Table 1:** Perioperative Characteristics of Patients with and without Postoperative TNEs (Transient Neurological Events)

Variable		Transient Neurological Events		***p***
	All Patients n = 312	Yes, n = 106	No, n = 206	
Age (y)	45.11 ± 9.96	43.14 ± 9.83	46.13 ± 9.89)	0.0119*
Male, n (%)	145(46.47)	63(59.43)	82(39.81)	0.001*
Body mass index (kg/m2)	24.2 ± 2.85	24.24 ± 2.96	24.19 ± 2.81	0.8849
Preoperative platelets(10^9/L)	190.13 ± 56.59	184.79 ± 54.09	192.88 ± 57.76	0.2325
Preoperative Hemoglobin (g/dL)	129.32 ± 14.18	132.68 ± 14.57	127.59 ± 13.69	0.0026*
Preoperative NA+(mmol/L)	141.4 ± 1.94	141.47 ± 1.92	141.36 ± 1.95	0.6434
Fasting blood glucose(mmol/L)	4.86 ± 0.88	4.73 ± 0.74	4.93 ± 0.94	0.0376*
Comorbidities, n (%)				
Hypertension	112 (35.9)	33 (31.13)	79 (38.35)	0.2081
Diabetes	19 (6.09)	4 (3.77)	15 (7.28)	0.2198
Stroke	39 (12.5)	19 (17.92)	20 (9.71)	0.0377*
Blood types, n (%)				0.0591
O	82 (26.28)	18 (16.98)	64 (31.07)	
A	86 (27.56)	33 (31.13)	53 (25.73)	
B	103 (33.01)	38 (35.85)	65 (31.55)	
AB	41 (13.14)	17 (16.04)	24 (11.65)	
Preoperative albumin(g/L)	40 ± 2.86	40.23 ± 2.99	39.88 ± 2.79	0.3008
Onset symptoms, n (%)				0.0463*
Ischemic	77(24.68)	35(33.02)	42(20.39)	
Hemorrhagic	50 (16.03)	14 (13.21)	36(17.48)	
Others	185 (59.29)	57(53.77)	128 (62.13)	
Intraoperative hypotension, n (%)	103(33.01)	45(42.45)	58(28.16)	0.0120*
Angiographic Suzuki Grade^**#**^, n (%)				0.0897
Grade III	73 (23.4)	31 (29.25)	42 (20.39)	
Grade IV	64 (20.51)	26 (24.53)	38 (18.45)	
Grade V	118 (37.82)	34 (32.08)	84 (40.78)	
Grade VI	57 (18.27)	15 (14.15)	42 (20.39)	
Duration of surgery(min)	341.34 ± 53.15	342.87 ± 53.66	340.55 ± 53	0.7163
Duration of anesthesia(min)	387.43 ± 52.39	388.84 ± 50.69	386.7 ± 53.35	0.7337
Infusion volume(ml)	4186.25 ± 1014.65	4191.98 ± 1090.68	4183.3 ± 975.98	0.9431
Postoperative Hemoglobin(g/dL)	111.58 ± 14.26	114.44 ± 14.16	110.1 ± 14.11	0.0106*
Postoperative platelets(10^9/L)	185.04 ± 50.96	185.35 ± 51.09	184.87 ± 51.02	0.938
Postoperative albumin(g/L)	33.13 ± 3.1	33.48 ± 3.34	32.95 ± 2.97	0.1508

Note: ^**#**^ Suzuki Grading System: Grade I: narrowing of ICA apex; grade II: initiation of moyamoya collaterals; grade III: progressive ICA stenosis with intensifification of moyamoya-associated collaterals; grade IV: development of ECA collaterals; grade V: intensifification of ECA collaterals and reduction of moyamoya-associated vessels; grade VI: total occlusion of ICA and disappearance of moyamoya-associated collaterals. * Indicates *P* < 0.05

**Fig. 2:**
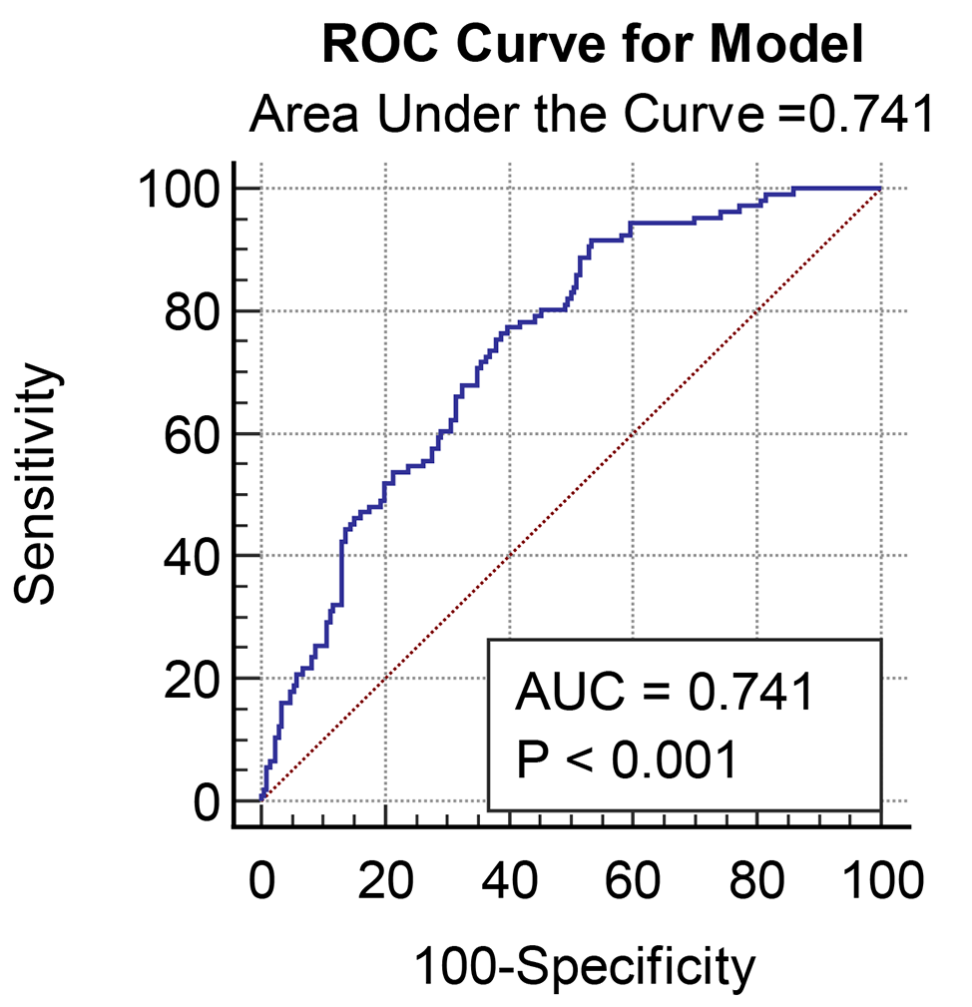
ROC curve of postoperative TNEs risk prediction model

In the regression model with TNEs as the outcome, only age, sex, fasting blood glucose, stroke status, blood type, onset symptoms, intraoperative hypotension and angiographic Suzuki grade were included in the final multivariate logistic regression. Males were more likely to suffer from postoperative TNEs (OR = 2.344,95% CI, 1.387, 3.961; *p* = 0.002). Preoperative ischemic presentation (OR = 1.849,95% CI, 1.005, 3.400; *p* = 0.048) and intraoperative hypotension (OR = 2.332, 95% CI, 1.350,4.030; *p* = 0.002) were associated with postoperative TNEs. Compared to patients with blood type O, patients with blood type B (OR = 2.239, 95% CI, 1.095, 4.580; p = 0.027), AB (OR = 2.938, 95% CI,1.197, 7.212; *p* = 0.019) and A (OR = 2.325, 95% CI, 1.096, 4.933; *p* = 0.028) had a significantly higher incidence of postoperative TNEs (Table 2). Blood types were still an independent risk factor for predicting postoperative TNEs with AUC = 0.741 (Fig. 2).

**Table 2:** Logistic Regression Analysis for Occurrence of Postoperative TNEs

Risk factors	Univariate logistic regression		Multivariable logistic regression	
	OR (95% CI)	*p* value	OR (95% CI)	*p* value
Age	0.970(0.947, 0.994)	0.013	0.979 (0.953, 1.006)	0.112
Male	2.215 (1.374, 3.571)	0.001	2.344(1.387, 3.961)	0.002*
BMI	1.006 (0.927, 1.092)	0.884		
Preoperative platelets	0.997 (0.993, 1.002)	0.232		
Preoperative Hemoglobin	1.026 (1.009, 1.044)	0.003		
Preoperative NA+	1.029 (0.912, 1.162)	0.642		
Fasting blood glucose	0.711 (0.499, 1.011)	0.058	0.715(0.498, 1.026)	0.068
Comorbidities				
Hypertension	0.727 (0.442, 1.196)	0.209		
Diabetes	0.499 (0.161, 1.544)	0.228		
Stroke	2.031 (1.031, 3.999)	0.040	2.010 (0.917, 4.404)	0.081
Blood types				
O	Reference		Reference	
A	2.214 (1.122, 4.370)	0.022	2.325 (1.096, 4.933)	0.028*
B	2.079 (1.076, 4.016)	0.029	2.239 (1.095, 4.580)	0.027*
AB	2.519 (1.118, 5.673)	0.026	2.938 (1.197, 7.212)	0.019*
Preoperative albumin	1.044 (0.962, 1.134)	0.301		
Onset symptoms				
Others	Reference		Reference	
Hemorrhagic	0.873 (0.437, 1.744)	0.701	1.024(0.484,2.167)	0.950
Ischemic	1.871(1.084, 3.232)	0.025	1.849(1.005, 3.400)	0.048*
Intraoperative hypotension	3.194(1.955, 5.218)	0.012	2.332(1.350, 4.030)	0.002*
Angiographic Suzuki Grade				
Grade III	Reference		Reference	
Grade IV	0.927 (0.469, 1.832)	0.827	1.247 (0.589,2.640)	0.563
Grade V	0.548 (0.297, 1.011)	0.054	0.552 (0.280,1.088)	0.086
Grade VI	0.484 (0.229, 1.025)	0.058	0.484 (0.214,1.098)	0.083
Duration of surgery	1.001 (0.996, 1.005)	0.715		
Duration of anesthesia	1.001 (0.996, 1.005)	0.733		
Infusion volume	1 (1, 1)	0.943		
Postoperative hemoglobin	1.022 (1.005, 1.039)	0.012		
Postoperative platelets	1 (0.996, 1.005)	0.938		
Postoperative albumin	1.057 (0.98, 1.14)	0.151		

* Indicates *P* < 0.05

## Discussion

Transient neurological events are commonly observed after surgical revascularization for MMD patients [14]. Previous studies have shown that these events will last for 7–10 days, which will increase the duration of hospitalization and the healthcare-related cost [15, 16]. However, the cause of TNEs in patients with MMD undergoing bypass surgery is still unclear. Our data show that gender, preoperative ischemic presentation, intraoperative hypotension and blood types were the risk factors of postoperative TNEs after revascularization surgery for MMD patients. Males were more likely to suffer from postoperative TNEs in our study. The relationship between gender and postoperative neurological events in patients with MMD was unclear. Study [17] has shown that female was independent risk factors for postoperative TNEs. However, A study has found that there is no gender difference in postoperative neurological outcomes in MMD [18]. A possible explanation for this may be related to different sex ratios of the selected research subjects. The presence of transient ischemic attack [19] and preoperative ischemic presentation [20] were identified as predictors of postoperative neurological outcomes, and our results were consistent with these research conclusions. Patients with the presence of transient ischemic attack were sensitive to intraoperative hemodynamic fluctuations, leading to the occurrence of postoperative TNEs.

Although study [21] has shown that the effect of intraoperative hypotension on perioperative stroke is unclear. Some studies [22, 23] have shown that intraoperative hypotension can be harmful to cerebral blood flow (CBF). Patients with moyamoya disease may be more sensitive to changes in CBF [24]. Intraoperative hypotension can aggravate insufficient cerebral microcirculation perfusion. The occurrence of intraoperative hypotension may lead to transient neurological events. Study has shown that intraoperative blood pressure fluctuations were independent risk factors for postoperative infarction after revascularization surgery in patients with moyamoya disease [25]. we also found intraoperative hypertension was associated with postoperative TNEs. There is no universal definition of intraoperative hypotension. We defined hypotension as a 20% reduction from baseline, study [26] suggested that this may be more appropriate for patients with chronic hypertension. Although our definition of hypotension is applicable to most patients, relative hypotension in few patients may be underrecognized.

Our study found a correlation between ABO blood type and the incidence of TNEs after surgical revascularization in patients with moyamoya disease. The incidence of TNEs in patients with blood type O was obviously lower than that in patients with blood type A, B or AB in our study. Studies have shown that non-O blood type individuals have increased risks of peripheral vascular disease, venous thrombosis, coronary heart disease, cancer, and ischemic stroke [27–31]. Differences in platelets, endothelial cells and coagulation factors among patients with different blood types may be the cause of this phenomenon [32, 33].

Genetic factors may be the reason that blood type is related to the incidence of postoperative TNEs. ABO blood groups are determined by the ABO gene [34]. As a genetic marker, the ABO blood group antigen is inherited and expressed in a wide variety of human cells and tissues, such as platelets, endothelial cells and sensory neurons [35, 36]. Some studies [37, 38] have shown that peripheral vasospasm is related to increased levels of VIII factor. Compared with other blood types, plasma levels of von Willebrand factor (vWF) and factor VIII were significantly decreased in patients with blood type O [39]. Low levels of factor VIII and vWF might be the reason for the relatively low incidence of postoperative TNEs after surgical revascularization for MMD patients with blood type O.

Nitric oxide (NO) regulates regional cerebral blood flow and correlates with the occurrence of ischemia [40, 41]. Studies [42] have shown that the reduction of contents of NO can inhibit endothelium-dependent vasodilatation function, leading to cerebral vasospasm. Nitric oxide (NO) regulates regional cerebral blood flow and correlates with the occurrence of ischemia [40, 41]. Studies [42] have shown that the reduction of contents of NO can inhibit endothelium-dependent vasodilatation function, leading to cerebral vasospasm. Local cortical hyperperfusion and watershed shift caused by vasospasm after surgical revascularization might be the cause of postoperative TNEs [43, 44]. NO may play a role in regulating the occurrence of vasospasm after revascularization surgery for MMD. Individuals with blood type B or AB were significantly less responsive to NO than individuals with blood type O or A [45]. NO reaction may be another reason for the relatively low incidence of TNEs in patients with blood type O compared with others.

There were several limitations in our study. First, our study was based on single center results, so selection bias is possible. Second, the study included an unequal distribution of patients with different blood types, which may have affected the results. Third, although many clinical factors were recorded in our study, the changes of postoperative blood pressure and other unknown confounders were not analyzed in this study. Last, Hemodynamic studies were mandatory to discuss TNEs after revascularization surgery for MMD. It remains uncertain which types of hemodynamic changes (hyperperfusion, hypoperfusion, watershed shift, and so on) result from ABO blood type. Further studies are required to verify which hemodynamic changes are affected by ABO blood type after surgical revascularization for Moyamoya disease.

## Conclusion

The incidence of postoperative TNEs was 34% in our study. Males, preoperative ischemic presentation, intraoperative hypotension were risk factors for postoperative TNEs. We also found a possible link between postoperative TNEs and ABO blood types after surgical revascularization for moyamoya patients.

## Data Availability

The datasets used and/or analysed during the current study are available from the corresponding author upon reasonable request.

## Abbreviations

MMD: Moyamoya disease
TNEs: transient neurological events
ROC curve: receiver operating characteristic curve
STA-MCA: superficial temporal artery-middle cerebral artery
NICU: neurosurgery intensive care unit
CBF: cerebral blood flow
NO: Nitric oxide
BMI: body mass index
SD: standard deviation

## References

[1] Kuroda S, Houkin K (2008). Moyamoya Disease: current concepts and future perspectives. Lancet Neurol.

[2] Kim JS (2016). Moyamoya Disease: Epidemiology, clinical features, and diagnosis. J Stroke.

[3] Lee S, Yun TJ, Yoo RE, Yoon BW, Kang KM, Choi SH, Kim JH, Kim JE, Sohn CH, Han MH (2018). Monitoring cerebral perfusion changes after revascularization in patients with Moyamoya Disease by using arterial spin-labeling MR Imaging. Radiology.

[4] Lotzke H, Jakobsson M, Brisby H, Gutke A, Hägg O, Smeets R, den Hollander M, Olsson LE, Lundberg M (2016). Use of the PREPARE (PREhabilitation, physical activity and exeRcisE) program to improve outcomes after lumbar fusion Surgery for severe low back pain: a study protocol of a person-centred randomised controlled trial. BMC Musculoskelet Disord.

[5] Hayashi K, Horie N, Suyama K, Nagata I (2012). Incidence and clinical features of symptomatic cerebral hyperperfusion Syndrome after Vascular Reconstruction. World Neurosurg.

[6] Kazumata K, Ito M, Tokairin K, Ito Y, Houkin K, Nakayama N, Kuroda S, Ishikawa T, Kamiyama H (2014). The frequency of postoperative Stroke in moyamoya Disease following combined revascularization: a single-university series and systematic review. J Neurosurg.

[7] Garvert L, Baune BT, Berger K, Boomsma DI, Breen G, Greinacher A, Hamilton SP, Levinson DF, Lewis CM, Lucae S et al. The association between genetically determined ABO blood types and major depressive disorder. Psychiatry Res. 2021;299. 10.1016/j.psychres.2021.113837 PMC8071927 33721783

[8] Simoni AH, Jerwiarz A, Randers A, Gazerani P (2017). Association between ABO blood types and pain perception. Somatosens Mot Res.

[9] Franchini M, Liumbruno GM, Lippi G (2016). The prognostic value of ABO blood group in cancer patients. Blood Transfus.

[10] Zhang H, Mooney CJ, Reilly MP (2012). ABO Blood groups and Cardiovascular Diseases. Int J Vascular Med.

[11] Unda SR, Vats T, Garza RDL, Cezaryirli P, Altschul DJ (2020). Role of ABO blood type in delayed cerebral ischemia onset and clinical outcomes after aneurysmal subarachnoid Hemorrhage in an ethnic minority urban population. Surg Neurol Int.

[12] Hamano E, Kataoka H, Morita N, Maruyama D, Satow T, Iihara K, Takahashi JC (2017). Clinical implications of the cortical hyperintensity belt sign in fluid-attenuated inversion recovery images after bypass Surgery for moyamoya Disease. J Neurosurg.

[13] Egashira Y, Yamauchi K, Enomoto Y, Nakayama N, Yoshimura S, Iwama T (2017). Disruption of Cortical Arterial Network is Associated with the severity of transient neurologic events after direct bypass Surgery in adult Moyamoya Disease. World Neurosurg.

[14] Fujimura M, Kaneta T, Mugikura S, Shimizu H, Tominaga T (2007). Temporary neurologic deterioration due to cerebral hyperperfusion after superficial temporal artery-middle cerebral artery anastomosis in patients with adult-onset moyamoya Disease. Surg Neurol.

[15] Fujimura M, Shimizu H, Mugikura S, Tominaga T (2009). Delayed intracerebral Hemorrhage after superficial temporal artery-middle cerebral artery anastomosis in a patient with moyamoya Disease: possible involvement of cerebral hyperperfusion and increased vascular permeability. Surg Neurol.

[16] Ogasawara K, Komoribayashi N, Kobayashi M, Fukuda T, Inoue T, Yamadate K, Ogawa A (2005). Neural damage caused by cerebral hyperperfusion after arterial bypass Surgery in a patient with moyamoya Disease: case report. Neurosurgery.

[17] Lu J, Zhao Y, Ma L, Chen Y, Li M, Chen X, Ye X, Wang R, Zhao Y (2019). Predictors and clinical features of transient neurological events after combined bypass revascularization for moyamoya Disease. Clin Neurol Neurosurg.

[18] Khan N, Achrol AS, Guzman R, Burns TC, Dodd R, Bell-Stephens T, Steinberg GK (2012). Sex differences in clinical presentation and treatment outcomes in Moyamoya Disease. Neurosurgery.

[19] Bao XY, Duan L, Li DS, Yang WZ, Sun WJ, Zhang ZS, Zong R, Han C (2012). Clinical features, Surgical Treatment and Long-Term Outcome in Adult patients with Moyamoya Disease in China. Cerebrovasc Dis.

[20] Zhao M, Deng XF, Zhang D, Wang S, Zhang Y, Wang R, Zhao JZ (2019). Risk factors for and outcomes of postoperative Complications in adult patients with moyamoya Disease. J Neurosurg.

[21] Wongtangman K, Wachtendorf LJ, Blank M, Grabitz SD, Linhardt FC, Azimaraghi O, Raub D, Pham S, Kendale SM, Low YH (2021). Effect of intraoperative arterial hypotension on the risk of Perioperative Stroke after noncardiac Surgery: a retrospective Multicenter Cohort Study. Anesth Analg.

[22] Gregory A, Stapelfeldt WH, Khanna AK, Smischney NJ, Boero IJ, Chen QY, Stevens M, Shaw AD (2021). Intraoperative hypotension is Associated with adverse clinical outcomes after noncardiac Surgery. Anesth Analg.

[23] Bijker JB, Persoon S, Peelen LM, Moons KGM, Kalkman CJ, Kappelle LJ, van Klei WA (2012). Intraoperative Hypotension and perioperative ischemic Stroke after general Surgery a nested case-control study. Anesthesiology.

[24] Kim SH, Choi JU, Yang KH, Kim TG, Kim DS (2005). Risk factors for postoperative ischemic Complications in patients with moyamoya Disease. J Neurosurg.

[25] Li JX, Zhao YH, Zhao M, Cao PH, Liu XJ, Ren H, Zhang D, Zhang Y, Wang R, Zhao JZ (2020). High variance of intraoperative blood pressure predicts early cerebral infarction after revascularization Surgery in patients with Moyamoya Disease. Neurosurg Rev.

[26] Meng L, Yu W, Wang T, Zhang L, Heerdt PM, Gelb AW (2018). Blood pressure targets in Perioperative Care. Hypertens (Dallas Tex: 1979).

[27] Goumidi L, Thibord F, Wiggins KL, Li-Gao RF, Brown MR, Vlieg AV, Souto JC, Soria JM, Ibrahim-Kosta M, Saut N (2021). Association between ABO haplotypes and the risk of venous Thrombosis: impact on Disease risk estimation. Blood.

[28] Tirado I, Mateo J, Soria JM, Oliver A, Martínez-Sánchez E, Vallvé C, Borrell M, Urrutia T, Fontcuberta J (2005). The ABO blood group genotype and factor VIII levels as Independent risk factors for venous thromboembolism. Thromb Haemost.

[29] He M, Wolpin B, Rexrode K, Manson JE, Rimm E, Hu FB, Qi L (2012). ABO blood group and risk of coronary Heart Disease in two prospective cohort studies. Arterioscler Thromb Vasc Biol.

[30] Amundadottir L, Kraft P, Stolzenberg-Solomon RZ, Fuchs CS, Petersen GM, Arslan AA, Bueno-de-Mesquita HB, Gross M, Helzlsouer K, Jacobs EJ (2009). Genome-wide association study identifies variants in the ABO locus associated with susceptibility to Pancreatic cancer. Nat Genet.

[31] Jaworek T, Xu H, Gaynor BJ, Cole JW, Rannikmae K, Stanne TM, Tomppo L, Abedi V, Amouyel P, Armstrong ND (2022). Contribution of common genetic variants to risk of early onset ischemic Stroke. Neurology.

[32] Jenkins PV, O’Donnell JS (2006). ABO blood group determines plasma von Willebrand factor levels: a biologic function after all?. Transfusion.

[33] Gallinaro L, Cattini MG, Sztukowska M, Padrini R, Sartorello F, Pontara E, Bertomoro A, Daidone V, Pagnan A, Casonato A (2008). A shorter von Willebrand factor survival in O blood group subjects explains how ABO determinants influence plasma von Willebrand factor. Blood.

[34] Yamamoto F, Clausen H, White T, Marken J, Hakomori S (1990). Molecular genetic basis of the histo-blood group ABO system. Nature.

[35] Franchini M, Liumbruno GM (2013). ABO blood group: old dogma, new perspectives. Clin Chem Lab Med.

[36] Liumbruno GM, Franchini M (2013). Beyond immunohaematology: the role of the ABO blood group in human Diseases. Blood Transfus = Trasfusione del sangue.

[37] Zhang H, Mooney CJ, Reilly MP (2012). ABO Blood groups and Cardiovascular Diseases. Int J Vascular Med.

[38] Zabaneh D, Gaunt TR, Kumari M, Drenos F, Shah S, Berry D, Power C, Hypponen E, Shah T, Palmen J (2011). Genetic variants associated with Von Willebrand factor levels in healthy men and women identified using the HumanCVD BeadChip. Ann Hum Genet.

[39] O’Donnell J, Laffan MA (2001). The relationship between ABO histo-blood group, factor VIII and Von Willebrand factor. Transfus Med.

[40] Castillo J, Rama R, Davalos A (2000). Nitric oxide-related brain damage in acute ischemic Stroke. Stroke.

[41] Rifkind JM, Nagababu E, Barbiro-Michaely E, Ramasamy S, Pluta RM, Mayevsky A (2007). Nitrite infusion increases cerebral blood flow and decreases mean arterial blood pressure in rats: a role for red cell NO. Nitric Oxide: Biology and Chemistry.

[42] Vellimana AK, Milner E, Azad TD, Harries MD, Zhou ML, Gidday JM, Han BH, Zipfel GJ (2011). Endothelial nitric oxide synthase mediates endogenous protection against subarachnoid hemorrhage-induced cerebral vasospasm. Stroke.

[43] Mukerji N, Cook DJ, Steinberg GK (2015). Is local hypoperfusion the reason for transient neurological deficits after STA-MCA bypass for moyamoya Disease?. J Neurosurg.

[44] Hayashi T, Shirane R, Fujimura M, Tominaga T (2010). Postoperative neurological deterioration in pediatric moyamoya Disease: watershed shift and hyperperfusion clinical article. J Neurosurgery-Pediatrics.

[45] Weimann J, Bauer H, Bigatello L, Bloch KD, Martin E, Zapol WM (1998). ABO blood group and inhaled nitric oxide in acute respiratory distress syndrome. Lancet.

